# Editorial Chit-Chat

**Published:** 1878-04

**Authors:** 


### Editorial Chit-Chat.

VOLUME XIV.—We are growing in years. Just fourteen years since the first number of this journal was issued.

CORRECTED—the title page. Now bind your BISTOURYS and have an excellent book of reference in the household.

A FINE HORSE.—Mr. Wm. J. Dounce, of our city, has become the owner of a very fast trotting horse, *how* fast, the boys will learn when they try to overhaul him on the road.

TRICHINÆ.—An entire family, consisting of two adults and four children, died from eating pork that was infected with trichinæ, at Youngstown, Ohio, recently. If people will eat pork, they should cook it most thoroughly, and so destroy these dangerous little worms. Not any pork on *our* plate, if you please.

PROTECTIVE TARIFF.—About the best article we have read upon this subject, short and to the point, was an editorial in the daily *Gazette* of this city under date of March 15th. The *Gazette* is right. Laborers would be much better off, in our opinion, if we had no protective tariff of any sort. We could make our statement good, did our space permit.

HORSE FRIGHTENERS.—Who's to blame for the papers of assorted sizes, and variegated hues, that are whirling and tossing about our streets, to the great fright of the horses ? Some one should remove them, but just who, we are unable to designate. Perhaps the Street Commissioner might be induced to call some one to his aid and have the crazy flying kites removed, if only his attention were called to them.

NO MORE DEADHEADS.—Many of our former city subscribers will miss their BISTOURY this year. Our subscription list is growing so rapidly that we cannot afford to increase our regular edition for the benefit of deadheads or those who are in arrears.

Our son, Thad S. Up de Graff, Jr., has taken charge of the city list, has revised it, and stricken off every name that is not paid up. He will have full control, and subscribers will do well to keep their accounts square with him if they desire to be retained upon our lists. The journal will be on sale at Hall Bros., as usual, price fifteen cents per copy.

THE TITLE PAGE.—A most provoking blunder occurred in the title page, sent out in the January number, in which physician was mis-spelled, notwithstanding the error had been twice corrected in the proof. The entire edition was run off before a copy reached us, so that there remained no remedy for the conspicuous blunder, which stuck out from the otherwise handsome page, like a sore thumb from a white napkin.

We send out a new title page, with this number, to take the place of the deformed one.

OUR JUNE VACATION.—By this time our readers have doubtless learned that we spend the month of June in the woods, not reaching home until July first, so that the next number of the BISTOURY will probably not be ready for delivery before July 15th. This is the only rest we take in the entire year, and we always rejoice when the April number is in the hands of the printer, for then the time is near at hand when we have that delightful experience with our good friend H. and other lovers of nature, on the beautiful mountain streams of the Lycoming region. Bye, bye !

THE DIFFERENCE.—Fritz had indulged himself in some roguish pleasure which he had been warned against. As usual, he forgot himself, and rendered chastisement necessary. And this is the conversation we overheard in the next room, while employed in our study : "Now, my boy, you have disobeyed again, how shall I punish you ? Will you go to bed for the balance of the day, or receive a whipping ?"

Fritz—"Who's to do the whipping, you or Pa ?"

Ma (with dire calamity portrayed in every feature of her countenance)—"I am sir !"

Fritz—"Well, I guess I'll take the whipping. But if Pa's to do it, by George, I'd rather go to bed !"

INGERSOLL.—We had read many comments of the press upon the lectures of Robert G. Ingersoll, most of them of the most sweeping condemnation of the man and his views, until we were almost brought to believe that his speeches were so blasphemous as to shock the sensibilities of anyone who held sacred the teachings of the christian religion, consequently that more pain than pleasure would come of listening to him. But, we have heard Mr. Ingersoll for ourself, and must testify that a more eloquent, instructive and profitable lecture was never delivered in our hearing. If there was any infidelity, any blasphemous utterances against the christian religion of to-day, we failed to hear them. The title of his lecture was "Skulls," or a plea for freedom of thought, honesty and candor of expression of our beliefs—whatever they may be. His views of the duties of the inmates of the household toward each other, portraying a model family government, abounded in beautiful gems of expression, the teachings of which can but leave a good impression in every community where it is heard. We commend Mr. Ingersoll and his lecture on Skulls.

---

THE PARK CHURCH CHOIR, of this city, a quartette of wonderfully sweet singers, have been solicited to give concerts in some of our neighboring towns. We hear of a gentleman who has offered to take them upon a concerting tour, so confident is he of their ability to charm any community with their melody. The quartette is composed of Mrs. A. F. Gibson, soprano, than whom no sweeter singer is upon the stage to-day ; Miss Nellie J. Perry, alto ; Mr. R. Parmenter, tenor, and Mr. W. H. Perry, basso and leader, while Mr. W. Cramer accompanies them upon the organ and piano.

If this party should consent to give concerts, we predict for them unbounded success.

---

"PARVULES."—Since so much interest has been manifested in testing the advantage of "small and frequently repeated doses of medicine," as recommended in Ringer's Handbook of Therapeutics, physicians have been looking for a convenient form in which to administer medicine after this theory and have the doses of equal and uniform value. Wm. R. Warner & Co., have been prompt to supply this want in the form of little sugar-coated pills, which they have named "parvules." These parvules contain appreciable doses of medicines, from a hundredth of a grain up to any size dose required, depending upon the nature of the drug employed. They are beautifully prepared medicines, of uniform strength and purity, and exactly adapted to the wants of the physician whether he desires to give small or large doses, for the dose must depend upon the number of the parvules given. These medicines are the most convenient that have yet been offered the profession and cannot but prove very popular among those physicians who dispense their own prescriptions.

---

ACORNS.—A corn we have. It has its abiding place upon the member next door to our great toe in the same row or block. Ache ! Whoever saw one of those abominable excrescences that did not ache ? Just pick your foot up in your lap and look at the wretched little thing—look squarely at the top of its round, hard head, while you pull your toes apart to get a chance at it. It does not look as though it *could* hurt so infernally, now does it ? But the moment shoe and stocking are replaced and one attempts to walk upon that foot, he is forced to acknowledge the corn—it hurts ; unless a fellow's half corned himself, how could it do otherwise ? But we cornered it to-day and went for it with a sharp knife, and carefully dissected it from its resting place, and threw it out the window, when a luckless rooster picked it up and swallowed the morsel. He has not sounded his cornopean since the corn exchange, but we are rid of our cornicle all the same—thank fortune.

---

CHILDREN'S EXPOSURE.—We notice that at some of our grammar schools, in this city, the children are not permitted to enter the building until a certain hour, and so rigidly does this rule seem to be enforced that the pupils are kept outside even in the rain, until the proper hour shall have arrrived. We do not know that all the schools enforce this law, but this morning, while riding by one of the school buildings, we noticed fifty or more little children standing in the rain, while the janitor stood in the door-way, watch in hand, seemingly counting the minutes when he would condescend to permit the little folks to enter the hall. As their garments must have been damp, if not wet, we regarded it a very dangerous rule that exposed the children to sickness likely to ensue from sitting, during the school hours, in their damp or wet garments.

Somebody is to blame in this matter.

---

PLANT FLOWERS.—Rake off the leaves, spade up the flower beds, and prepare for a grand display of blossoms during the summer days, now coming. Do not waste your time over vegetables, unless your garden space is abundant and your time unlimited. You can buy more vegetables for your table, for twenty-five cents, than you can raise for a dollar and a half's worth of labor; while flowers,—O, the flowers !—why, they are a constant delight, and repay your labor ten-fold. Have a pansy bed, as large a one as you have room for, and amuse yourself in watching their pretty little heads as they rapidly come into bloom. For ten cents you can cultivate more pansies, with greater variety of faces than you can find among the babies of your neighborhood. And then they do look so saucy and knowing when you come to look upon them in the early Spring morning, when the sun causes them to raise their pretty heads and fairly laugh at you through the crystal moisture that sparkles over their pretty faces. Who could spend time over the cultivation of a crooked squash, a wormy radish, or a long-tailed beet, when flowers of beauty and fragrance can be had at much less labor and expense ? Give the pansies, phlox, asters, sweet alyssum, candytuft and verbenas a chance. They are so inexpensive and yet so grandly beautiful that, we have even seen a jackass rabbit stop and admire them and make a leap to clear the bed, rather than injure one of their beautiful petals.

---

SPRING TIME, with its buds, blossoms, green grass and wild flowers, has arrived—hurrah ! Hold on a minute, for, with it has also come pestilence and sickness, unless considerable attention has been paid to the premises upon which we have our abiding places. Look, therefore, to your back-yards—remove the bones, pealings and other decomposing vegetable and animal matter that has accumulated there during the winter. Rake and scrape the yard clean. Sprinkle lime where slops have accidentally fallen. Clean out and whitewash your cellars, and so prepare for a healthful summer campaign. Remember that typhoid fever, diphtheria and kindred complaints are directly traced to dirty yards, foul cellars, and defective cesspools.

---

THAT "GREAT ENGLISH PHYSICIAN."—Dr. Henry A. Burner, formerly of Buffalo, N. Y., and Erie, Pa., who recently arrived in New Haven and commenced practice, was arrested, with his agent, Anthony Bihler, yesterday, on the charge of trying to defraud Caleb T. Hubbard, a resident of the city and a brother of S. H. Hubbard, managing editor of the Hartford *Courant*, out of $100 as pay for removing a tape-worm which Mr. Hubbard says was never removed, and both were held in $500 apiece. The doctor (?) has also been sued by Mr. Hubbard for $12,000 damages for malpractice in the case.—*Springfield Republican.*

So it seems the great English physician is still at his old tricks—removing tape-worms at a hundred dollars a head, and supplying the animals himself. It is unfortunate for the doctor(?)that now and then he encounters a chap sharp enough to detect his little trick at legerdemain.

---

REUBEN.—Our readers, we are sure, will welcome back to the BISTOURY one of our former contributors, Mr. Ausburn Towner, the popular author. More than thirteen years ago, when the venture of publishing this journal was first discussed among a coterie of congenial spirits,—Ben. H. Pratt, Will Potter, Ausburn Towner and others,— considerable time was spent in the selection of a name that the new quarterly was to bear. Mr. Towner suggested THE BISTOURY, and stood godfather to the christening of a journal whose success has outstripped the anticipations of us all. In reading "Reuben," we trust our readers will not omit the foot-notes,—they are crammed full of interesting matter. The better to appreciate the title of the paper, it must be remembered that Reuben is accredited with having been a very unstable and changeable sort of chap.

---

THE PHONOGRAPH.—Really the most important invention that has occurred in the last century is the discovery of the means of reproducing sound. Mr. Edison exhibited the first machine he ever constructed, in this city, recently, to the surprise and delight of our scientific men. The machine is a very simple arrangement, consisting of a vibrating disk of thin sheet iron, from which projects a point of metal that impresses every vibration of the disk upon a sheet of tin-foil that is spread evenly over a grooved cylinder. As the point rests immediately over the groove the slightest motion of the point makes an indentation in the tin-foil. While the voice sets the disk to vibrating, the cylinder is turned by means of a crank, so that every sound has an indentation of more or less depth and length, depending upon the character of sound made. To reproduce it, the cylinder is returned to the starting point, when a trumpet is placed over the diaphragm, when, again turning the cylinder, the point is moved by the depressions in the tin-foil, causing the disk to vibrate in the same manner as when the voice agitated it, when first uttered. By this arrangement, a speech delivered to-day, can be reproduced tomorrow, or next year, or a hundred years hence. All that is now necessary to hear how our great orators and statesmen of to-day talked, a hundred years hence, is to have the impression of their speeches taken, and grind them out to an audience upon demand.

We will soon have talking dolls, singing artificial birds, clocks that will *tell* the time of day, and a multiplicity of talking things. Concerts, given by the great artists, in our larger cities, can be reproduced in the small villages or in our own parlors at a trifling cost. In short, the capabilities of the phonograph are almost beyond credence, and will give us many new developments to wonder over and talk about in the near future.

